# The Status of the Quality Control in Neuroimaging Studies of Acupuncture Analgesia

**DOI:** 10.1155/2020/8502530

**Published:** 2020-09-21

**Authors:** Zhen Gao, Gaofeng Liu, Jing Zhang, Laixi Ji

**Affiliations:** ^1^Acupuncture and Tuina School/The 3rd Teaching Hospital, Chengdu University of Traditional Chinese Medicine, Chengdu, Sichuan, China; ^2^Graduate Faculty, Tianjin University of Traditional Chinese Medicine, Tianjin, China; ^3^Affiliated Hospital of Shanxi University of Traditional Chinese Medicine, Taiyuan, Shanxi, China; ^4^Shanxi University of Traditional Chinese Medicine, Jinzhong, Shanxi, China

## Abstract

Neuroimaging technology is an important technology used to explore the neural mechanisms of acupuncture analgesia. In this study, we extracted original studies published in Chinese and English focusing on the use of neuroimaging technology to explore the mechanisms of acupuncture analgesia from PubMed, Cochrane Central Register of Controlled Trials (CENTRAL), EMBASE, Web of Science, and CNKI databases from January 1999 to August 2020. The extracted data were statistically analyzed in terms of year of publication, country, experimental design, and quality control approaches used, sample size, characteristics of participants, acupuncture operation, and other information. Analysis of the literature revealed that international cooperation promotes scientific research. Flexible experimental design can better explain the mechanism of acupuncture analgesia. Reasonable sample size, strict participant inclusion criteria, and standard acupuncture practices are essential for repeatability of conclusions. These findings show that attention should be paid to quality control in future research to improve the reliability of research on acupuncture analgesia.

## 1. Introduction

The International Pain Research Association (IPRA) defines pain as an unpleasant feeling and emotional experience associated with actual or potential tissue damage. It has huge implications on individuals and society [1]. Research on pain has been intensified in the past decades achieving encouraging results. Acupuncture as an integral part of complementary and alternative therapy has been widely used for pain treatment due to its good efficacy and safety [2, 3]. However, its therapeutic mechanism remains elusive. Before the advent of neuroimaging technology, the research on the relationship between acupuncture and central nervous system has been limited due to the lack of noninvasive research methods. The development of functional magnetic resonance imaging (fMRI), positron emission computed tomography (PET), single-photon emission computed tomography (SPECT), electrography (EEG), magnetoencephalography (MEG), and other technologies has made it possible to study the mechanisms of acupuncture analgesia in the human nervous system. Notably, results of previous imaging studies vary with the pre-experimental research settings. For example, results from similar studies may be quite different and even contradictory [4, 5]. Therefore, strict quality control systems should be put in place to guarantee high reliability and repeatability of research conclusions.

This study aimed to analyze the current status of neuroimaging research on acupuncture analgesia in terms of quality control and research design by comparing original articles published in Chinese with those published in English from 1999 to 2020 to provide a reference for future research.

## 2. Methods

### 2.1. Searching Strategy

We searched the original articles published during January 1, 1999, to August 6, 2020, on PubMed, Cochrane Central Register of Controlled Trial (CENTRAL), EMBASE, and Web of Science using the following MeSH terms and search strategies: (“Acupuncture” [Mesh] OR “Acupuncture Therapy” [Mesh] OR “Acupuncture, Ear” [Mesh] OR “Acupuncture Points” [Mesh] OR “Acupuncture Analgesia” [Mesh]) AND (“pain” [Mesh]) AND (“Neuroimaging” [Mesh] OR “Functional Neuroimaging” [Mesh] OR “Functional MRI” [Mesh] OR “PET” [Mesh] OR “EEG” [Mesh] OR “MEG” [Mesh] OR “SPECT” [Mesh] OR “TCD” [Mesh]). Also, we searched China National Knowledge Infrastructure (CNKI) for articles from January 1, 1999, to August 6, 2020, in Chinese language by the title/abstract/keyword search. The detailed search terms and search strategies are as follows: (“zhenci” OR “dianzhen” OR “shouzhen” OR “zhenjiu” OR “touzhen” OR “erzhen” OR “wanhuaizhen”) AND (“tengtong” OR “zhentong”) AND (“Functional MRI” OR “functional magnetic resonance imaging” OR “PET” OR “Positron emission tomography” OR “EEG” OR “electroencephalography” OR “MEG” OR “magnetoencephalography” OR “SPECT” OR “Single-Photon Emission Computed Tomography” OR “TCD” OR “Transcranial Doppler”).

We screened the bibliographies of identified trials and reviewed articles for further potentially relevant publication. Subsequently, we screened the full texts and assessed whether these articles met the inclusion criteria.

### 2.2. Inclusion and Exclusion Criteria

The articles would be included, if they were (1) original articles; (2) neuroimaging study of acupuncture analgesia in human on human beings; (3) published in English or Chinese; and (4) published from January 1, 1999, to August 6, 2020.

**Figure 1:**
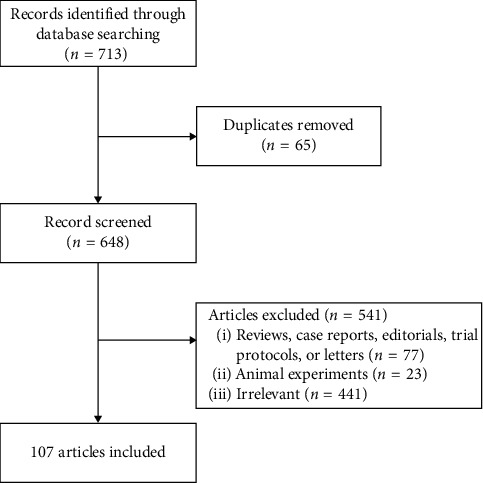
The flowchart of literature search and screening process.

The articles would be excluded if they were (1) reviews or editorials or trial protocols; (2) neuroimaging studies on animals; (3) unable to obtain full text; or (4) duplicate articles. The flow chart of article selection is shown in Figure 1.

### 2.3. Data Extraction and Analysis

We extracted the data including the year of publication, nationality of the author, characteristics of the participant (patients or the health, gender, handedness, diseases, emotional state, and acupuncture experience), sample size, acupuncture intervention (method of intervention, acupoint selection, manipulation procedure, Deqi/needle sensation, and acupuncturist), outcome assessment, neuroimaging technology, and ethical review. Data analysis was conducted after data extraction.

## 3. Results

107 original articles were included.

### 3.1. Annual Distribution of the Studies

**Figure 2:**
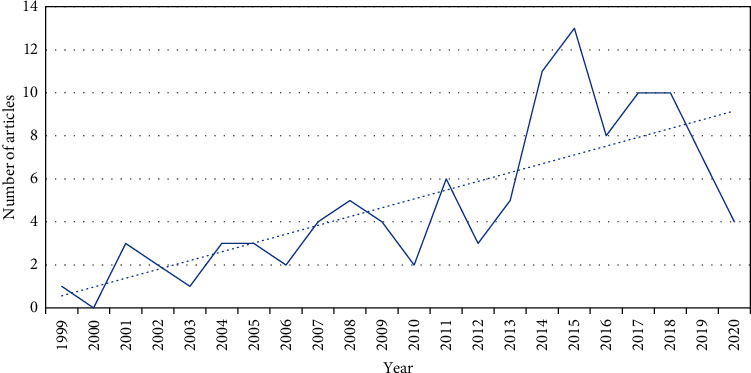
Annual distribution of acupuncture analgesia neuroimaging research.

In the past 20 years, the overall trend of neuroimaging studies on acupuncture analgesia has been increasing, which reached its peak in 2015. Its annual distribution changes are shown in Figure 2.

### 3.2. Nationality Distribution of the Studies

**Figure 3:**
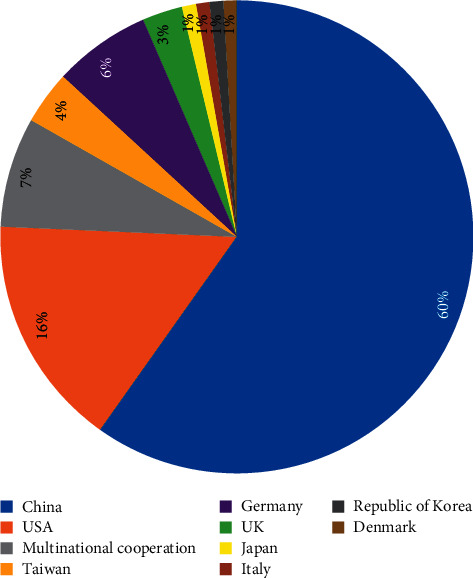
The nationality distribution of acupuncture analgesia neuroimaging research.

Most of the studies were conducted in China (64 studies), followed by the United States (17 studies) and Germany (7 studies). Neuroimaging studies on acupuncture analgesia have also been published in Taiwan (4 studies), the United Kingdom (3 studies), Republic of Korea (1 study), Japan (1 study), Denmark (1 study), and Italy (1 study). Four of the studies were conducted by the United States and Republic of Korea, three was conducted in cooperation with China and the United States, and one was the cooperation between Denmark and Italy (Figure 3).

### 3.3. Sample Size

The average sample size of these studies was 40 participants, and the maximal and minimal sample sizes were 160 participants and 6 participants, respectively. For the studies performed on patients, the average sample size was 48 participants, while the maximal and minimal sample sizes per group were 80 participants and 6 participants, respectively. For those performed on healthy subjects, the average sample size was 23, and the maximal and minimal sample sizes per group were 40 participants and 3 participants, respectively.

### 3.4. Status of Participants

#### 3.4.1. Classification of Participants

Thirty-three studies were performed on healthy subjects. 60 studies were performed on patients, and 14 studies recruited both healthy subjects and patients, involving 18 diseases (Table 1).

#### 3.4.2. Gender

Ninety-five studies described the gender of the participants (38.70% male and 61.30% female). 12 studies did not mention the gender of the participants.

#### 3.4.3. Handedness

Fifty-five studies asked for the right-hand participants in inclusion criteria, accounting for 51.40% of the total study population.

#### 3.4.4. Acupuncture Experience

Thirty-seven studies described the acupuncture experience of participants. Among these articles, 17 studies described the participants as acupuncture naive.

#### 3.4.5. Emotional State

The psychological assessment on the participants was performed in 22 studies. The Self-Rating Anxiety Scale (SAS) was the most used, used in 7 studies. The Self-Rating Depression Scale (SDS) was used in 5 studies. Mini-Mental State Examination (MMSE), the Hamilton Depression Scale (HAMD), and the Pittsburgh Sleep Quality Index (PSQI) were used in 2 studies. The Beck Depression Inventory (BDI) and the State and Trait Anxiety Questionnaire (STAI) were used in 1 study, respectively. 9 studies have excluded the participants with claustrophobia.

### 3.5. Acupuncture Intervention

#### 3.5.1. Acupuncture Modalities

Sixty-six studies chose manual acupuncture as the intervention method. 29 studies chose electroacupuncture as the intervention method. The transcutaneous electric acupoint stimulation was performed in 6 studies, and auricular acupuncture was performed in 2 studies. Besides, 4 studies used at least two methods.

#### 3.5.2. Acupoint Selection

Forty-seven studies were selected specific acupoint for acupuncture, and 60 studies used standardized and semistandard acupuncture treatment. In the selection of specific acupoints, the top 3 acupoints are LI 4 (17), ST 36 (10), and SP 6 (7) (Table 2). Among them, LI 4 (12), ST 36 (10), and LR 3 (4) were mostly used for acupuncture in healthy subjects.

#### 3.5.3. Manipulation Procedure

Except 10 studies which did not describe the acupuncture manipulation, the other 97 studies described the manipulation procedure of acupuncture.

#### 3.5.4. Qualification of Acupuncturists

Forty-seven studies mentioned the qualification of acupuncturists.

#### 3.5.5. Deqi (Needle Sensation)

Seventy-three studies required Deqi (needle sensation) during acupuncture stimulation. The 10-point Visual Analogue Scale (VAS), 0–10 Deqi Score, 11-point Numerical Pain Rating, Numerical Rate Scale (NRS), Acupuncture Sensation Scale (SASS), Needle Sensation Questionnaire (NSQ), and the Massachusetts General Hospital Acupuncture Sensation Scale (MASS) were used to evaluate the needle sensation in 25 studies.

### 3.6. Outcome Assessment

The outcome assessment on the participants was performed in 35 studies. The Visual Analogue Scale (VAS) (50 studies), McGill Pain Questionnaire (MPQ) (6 studies), Duration of pain (8 studies), PROMIS-29 pain interference (2 studies), Numeric Rating Scale (NRS) (2 studies), Brief Pain Inventory (BPI) (1 study), and pain threshold detector (1 study) were used.

### 3.7. Neuroimaging Technology

**Figure 4:**
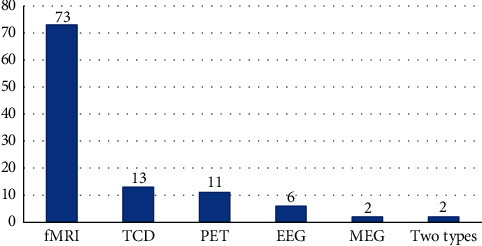
The techniques used in acupuncture analgesic neuroimaging studies. MRI: magnetic resonance imaging; TCD: transcranial doppler; PET: positron emission tomography; EEG: electroencephalography; MEG: magnetoencephalography.

73 studies used fMRI to investigate the cerebral responses to acupuncture stimulation, followed by TCD (13 studies) and PET (11 studies) studies. 2 studies used the combination of two imaging technologies (fMRI and EEG/fMRI and PET). The application of the techniques in neuroimaging studies of acupuncture analgesia is shown in Figure 4.

#### 3.7.1. fMRI Scanning Process

**Figure 5:**
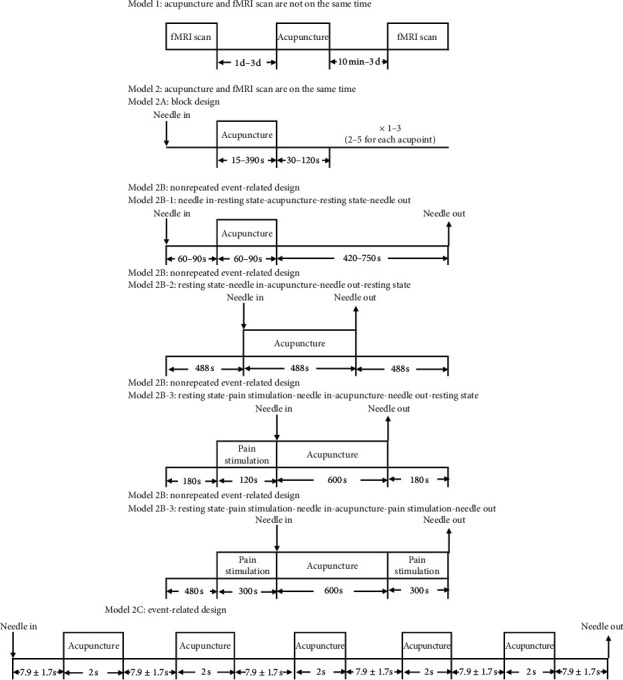
The models of fMRI scan.

The fMRI scanning processes can be divided into two modes: (1) acupuncture and fMRI scan are not on the same time (41 studies) (Figure 5, Model (1)) and (2) acupuncture and fMRI scan are on the same time (34 studies) (Figure 5, Model (2)).

According to the different scan sequence designs, fMRI can be divided into block design (Figure 5, Model 2A), nonrepeated event-related design (Figure 5, Model 2B), event-related design (Figure 5, Model 2C), and a combination of the two designs.

#### 3.7.2. PET Scanning Process

**Figure 6:**
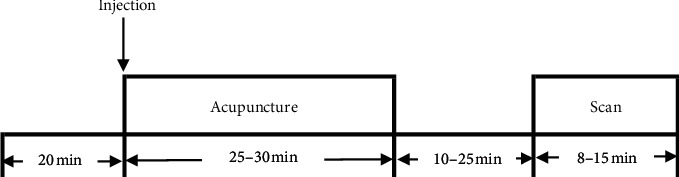
The models of PET scan.

Among the imaging agents, fluorine-18 fluorodeoxyglucose (18F-FDG) (7 studies) was most commonly used in the PET scanning, 3 studies used H215O, and the other 2 studies used 11C-carfentanil or [11C] diprenorphine, respectively. Most studies were conducted using the model in Figure 6.

#### 3.7.3. EEG Scanning Process

**Figure 7:**
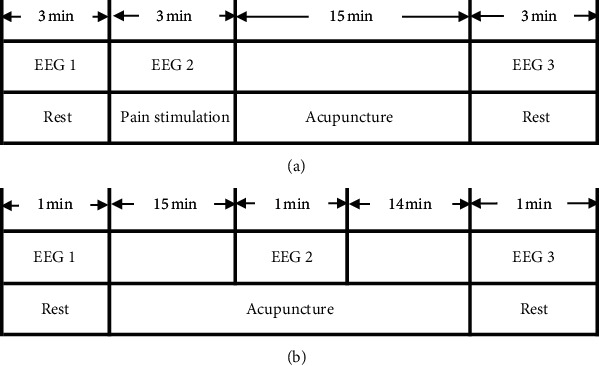
The models of EEG scan. (a) Model 1: EEG scan gives pain stimulation. (b) Model 2: EEG scan does not give pain stimulation.

According to whether to give pain stimulation, EEG scanning can be divided into two modes: giving pain stimulation (Figure 7(a)) and not giving pain stimulation (Figure 7(b)).

#### 3.7.4. MEG Scanning Process

**Figure 8:**
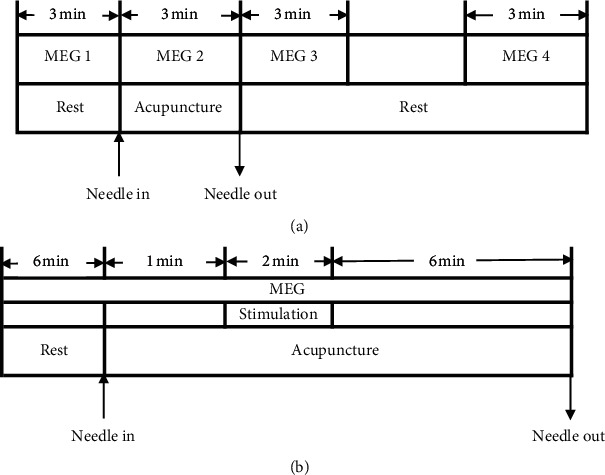
The models of MEG scan. (a) Model 1: MEG intermittent scan. (b) Model 2: MEG continuous scan.

According to the time of MEG scanning, MEG scanning can be divided into two modes: intermittent scanning (Figure 8(a)) and continuous scanning (Figure 8(b)).

### 3.8. Ethical Review

Seventy-one studies have mentioned the ethical review in the study.

## 4. Discussion

Acupuncture is a common method of analgesia, and the study of central mechanism has become a hot spot in neuroimaging. To improve the repeatability and reliability of results, it is necessary to employ rigorous and robust methods. This study analyzed the status of quality control systems applied in acupuncture analgesia research using neuroimaging methods for the first time to generate new ideas for future studies.

### 4.1. Sample Size

The size of the sample size directly affects the statistical power of the results. Our statistical analyses showed that the sample size of neuroimaging studies on acupuncture analgesia varied from 6 to 160 participants, with significant differences in results. This may be ascribed to the high cost of imaging technology, potential radioactivity (PET), and irregular experimental design. Regarding the sample size, some investigators suggested that 12–15 subjects represent a reasonable sample size to stably measure cerebral responses in fMRI studies [6]. Others held the view that a minimum sample size of 20 participants per group should be used to obtain 80% power [7]. It has also been suggested that smaller samples will result in a higher false negative rate [8], which will require multiple comparison correction to eliminate false positive rates [9]. There is some consensus among some researchers that to obtain repeatable results and to achieve the stable statistical power, a larger sample size is paramount (at least 40 participants per group) [10].

### 4.2. Status of Participants

This study revealed that the majority of neuroimaging studies (69.16%) were performed on patients. According to the theory of traditional Chinese acupuncture, the effect of acupuncture is significant in individuals suffering from certain diseases, and its effects are negligible in healthy people. Therefore, it is advisable that neuroimaging studies on acupuncture analgesia should be carried out on patients [11, 12]. Migraine is a chronic vascular disease with multiple subtypes. Our analysis showed that half of the studies did not study its specific subtypes, ignoring possible differences in brain function and structure among patients with different disease subtypes [13, 14]. In addition, musculoskeletal pain, such as low back pain, neck pain, and fibromyalgia, have attracted substantial research attention.

Previous studies have revealed gender differences in the human brain [15, 16], not only anatomically [17] but also in the fields of behavior and cognition [18, 19]. Moreover, because of different handedness, there are differences in the structure of the brain area [20, 21], and the right-handed choice has always been favored. Some studies suggest that pain sensitivity may vary with different psychosocial factors [22]. In our study, we found that participants in 15.89% of the studies were acupuncture naïve, and 20.56% of the studies conducted psychological assessments on the participants. To increase homogeneity among studies, acupuncture experience and psychological state of the participants should be considered.

### 4.3. Acupuncture Intervention

We found that 61.68% of the studies chose the most traditional manual acupuncture as the intervention method, but the consistency of the operation process compared with electroacupuncture is still unproven. The most commonly studied acupuncture points are LI 4, ST 36, and SP 6, because they are considered to have good analgesic effects. In addition, to improve quality control, 90.65% of the studies described the operation process of acupuncture, including the insertion depth, manipulation, and retention time and 43.93% of the studies place restrictions on the qualification of the acupuncturist to achieve the desired effects of acupuncture.

Deqi is believed to be closely related to the efficacy of acupuncture, especially its analgesic effect. Moreover, Deqi influences the success of acupuncture anesthesia. In our study, 68.22% of the studies required Deqi, but only 23.36% of the studies used a questionnaire to assess the acupuncture sensation. Similarly, the results of acupuncture were assessed based on a subjective questionnaire. Only one study used objective tool for assessment. The definition of the degree of pain depends on many factors, and objective indicators seem to reflect the treatment effect more realistically.

### 4.4. Neuroimaging Technology

#### 4.4.1. fMRI Scanning Process

In our study, fMRI scanning processes were divided according to 2 aspects: (1) whether the acupuncture stimulation was performed at the same time as the fMRI scan. According to this condition, the scanning processes were divided into two models: Model 1, acupuncture and fMRI scan were not performed at the same time and Model 2 in which acupuncture and fMRI scan were performed at the same time, (2) based on different scanning sequence, Model 2 was divided into 4 types: Model 2A is scanning with block design, Model 2B is scanning with nonrepeated event-related design, Model 2C is scanning with event-related design.

The main feature of Model 1 is that scanning was performed completely during the resting state. This model is mainly used to observe the changes of brain response characteristics and has little interference during the scanning process compared with Model 2. The main feature of Model 2 is that scanning is conducted in the state of the task. Model 2A presents several separate stimulation sequences composed of tasks of the same nature to participants alternately and is now the most widely used method of acupuncture analgesia, due to its high signal detection ability. However, it can only estimate the cortical activation pattern caused by a certain block of tasks and cannot observe the effect of separate events; hence, it may produce expected effects due to the fixed sequence position of the block. Model 2B is designed to provide only one acupuncture stimulation during one scanning process and perform scanning with the needle (Model 2B-1) or without the needle (Model 2B-2). This design can effectively reduce the interference of the persistent effect of acupuncture on the resting block in the block design. Model 2C selects the events of interest to the researchers as stimulus sequences in an isolated way to trigger short-term cortical activation, which not only overcomes the limitation of block design that cannot randomly present stimuli but also effectively restores the signals related to the event.

#### 4.4.2. TCD Scanning Process

As the second most commonly used neuroimaging technology, TCD can measure changes in brain hemodynamics before and after treatment. However, since it was determined by visual waveforms and auditory feedback waves of ultrasonic signals, the accuracy of repeated measurements of blood flow velocity was reduced.

#### 4.4.3. PET Scanning Process

PET scanning for acupuncture analgesia is usually performed after acupuncture to examine the immediate effect of acupuncture. Previous studies have suggested that acupuncture after injection of the imaging agent can avoid false positive results [23], which is in agreement with our findings. However, the duration of acupuncture after injection has not been determined. Based on the half-life of imaging agent, some studies have proposed that acupuncture should be performed 30–45 minutes after injection [11].

#### 4.4.4. EEG Scanning Process

According to whether pain stimuli are generated, EEG is divided into two categories: Model 1 which is a study performed on healthy participants. It gives pain stimuli, such as injections of medicines, and scans after acupuncture. Model 2 is a study performed on patients. It chooses to scan at different periods before, during, and after acupuncture. They are used to observe the effect of acupuncture treatment.

#### 4.4.5. MEG Scanning Process

According to the timing of the MEG scan, the MEG scan can be divided into two categories: Model 1 is an intermittent scan, which collects the signal before acupuncture, signal during acupuncture, and the two signals during the two periods after the acupuncture, and observes the changes in brain magnetic field activity at different times. Model 2 is a continuous scan, which completely collects the signals before and during acupuncture, and analyzes the change patterns of different oscillation signal activities of the brain.

### 4.5. Author Nationality and Ethical Review

In this study, we found that China, as the birthplace of acupuncture, was the country with the highest number of publications in neuroimaging research on acupuncture analgesia (59.81%), but only 3 studies were from international cooperation teams. To promote the development of this field, international cooperation is advocated. Furthermore, to ensure that the safety, health, and rights of participants are protected, ethical review should be mandatory.

## 5. Conclusion

This study analyzes the current status of neuroimaging research on acupuncture analgesia and describes the experimental methods and technologies involved. Our results serve as important reference for future research.

## Figures and Tables

**Table 1:** The diseases involved in neuroimaging studies of acupuncture analgesia.

Disease	Number of studies
Migraine	27
Low back pain	14
Knee osteoarthritis	6
Carpal tunnel syndrome	4
Primary dysmenorrhea	4
Cervical spondylosis	3
Headache	2
Postoperative pain	2
Sciatica	2
Peripheral facial paralysis	1
Irritable bowel syndrome	1
Shoulder pain	1
Thalamic pain	1
Fibromyalgia	1
Rheumatoid arthritis	1
Endometriosis	1
Pancreatitis	1
Phalangeal osteoarthritis	1
Unspecified	1

**Table 2:** The top 10 acupuncture analgesia points.

Acupoint	Number of uses
LI 4	17
ST 36	10
SP 6	7
GB 34	4
GB 39	4
LR 3	4
LI 3	4
ST 44	3
SJ 5	2
GB 40	2
PC 6	2
SI 3	2

## Data Availability

The raw data used to support the study can be directly obtained from the PubMed, Cochrane Central Register of Controlled Trials (CENTRAL), EMBASE, Web of Science, and CNKI.

## Supplementary Materials

Supplementary Materials The table contains detailed information about the review.

## References

[1] Lee G. I., Neumeister M. W. (2020). Pain: pathways and physiology. *Clinics in Plastic Surgery*.

[2] Kelly R. B., Willis J. (2019). Acupuncture for pain. *American Family Physician*.

[3] Hamlin A. S., Robertson T. M. (2017). Pain and complementary therapies. *Critical Care Nursing Clinics of North America*.

[4] Liu Z., Wei W., Bai L. (2014). Exploring the patterns of acupuncture on mild cognitive impairment patients using regional homogeneity. *PLoS One*.

[5] MacPherson H., Green G., Nevado A. (2008). Brain imaging of acupuncture: comparing superficial with deep needling. *Neuroscience Letters*.

[6] Hayasaka S., Peiffer A. M., Hugenschmidt C. E., Laurienti P. J. (2007). Power and sample size calculation for neuroimaging studies by non-central random field theory. *Neuroimage*.

[7] Desmond J. E., Glover G. H. (2002). Estimating sample size in functional MRI (fMRI) neuroimaging studies: statistical power analyses. *Journal of Neuroscience Methods*.

[8] Poldrack R. A. (2012). The future of fMRI in cognitive neuroscience. *Neuroimage*.

[9] Lu W., Dong K., Cui D., Jiao Q., Qiu J. (2019). Quality assurance of human functional magnetic resonance imaging: a literature review. *Quantitative Imaging in Medicine and Surgery*.

[10] Chen X., Lu B., Yan C.-G. (2018). Reproducibility of R-fMRI metrics on the impact of different strategies for multiple comparison correction and sample sizes. *Human Brain Mapping*.

[11] He Z., Hou L., Sun R. (2019). The status of the acupuncture mechanism study based on PET/PET-CT technique: design and quality control. *Evidence-Based Complementary and Alternative Medicine*.

[12] Qiu K., Jing M., Sun R. (2016). The status of the quality control in acupuncture-neuroimaging studies. *Evidence-Based Complementary and Alternative Medicine*.

[13] Ma T., Zeng F., Li Y. (2015). Which subtype of functional dyspepsia patients responses better to acupuncture? A retrospective analysis of a randomized controlled trial. *Complementary Medicine Research*.

[14] Potkin S. G., Alva G., Fleming K. (2002). A PET study of the pathophysiology of negative symptoms in schizophrenia. *American Journal of Psychiatry*.

[15] Hu Y., Xu Q., Li K. (2013). Gender differences of brain glucose metabolic networks revealed by FDG-PET: evidence from a large cohort of 400 young adults. *PLoS One*.

[16] Racine M., Tousignant-Laflamme Y., Kloda L. A., Dion D., Dupuis G., Choinière M. (2012). A systematic literature review of 10 years of research on sex/gender and experimental pain perception—part 1: are there really differences between women and men?. *Pain*.

[17] Raz N., Gunning-Dixon F., Head D., Rodrigue K. M., Williamson A., Acker J. D. (2004). Aging, sexual dimorphism, and hemispheric asymmetry of the cerebral cortex: replicability of regional differences in volume. *Neurobiology of Aging*.

[18] Wang L., Shen H., Tang F., Zang Y., Hu D. (2012). Combined structural and resting-state functional MRI analysis of sexual dimorphism in the young adult human brain: an MVPA approach. *Neuroimage*.

[19] Crespo-Facorro B., Roiz-Santiáñez R., Pérez-Iglesias R. (2011). Sex-specific variation of MRI-based cortical morphometry in adult healthy volunteers: the effect on cognitive functioning. *Progress in Neuro-Psychopharmacology and Biological Psychiatry*.

[20] Damore D., Rutledge J., Pan S., Knotek N., Ramundo M. (2009). Handedness effects on procedural training in pediatrics. *Clinical Pediatrics*.

[21] Solodkin A., Hlustik P., Noll D. C., Small S. L. (2001). Lateralization of motor circuits and handedness during finger movements. *European Journal of Neurology*.

[22] Racine M., Tousignant-Laflamme Y., Kloda L. A., Dion D., Dupuis G., Choinière M. (2012). A systematic literature review of 10 years of research on sex/gender and pain perception—part 2: do biopsychosocial factors alter pain sensitivity differently in women and men?. *Pain*.

[23] Liu M. L., Lan L., Zeng F., Li X.-z., Liu X.-g., Liang F.-r. (2010). Quality control of the research on mechanisms of acupuncture therapy by using PET-CT imaging techniques. *Zhen Ci Yan Jiu*.

