# Supplementary material for: The Status of the Quality Control in Neuroimaging Studies of Acupuncture Analgesia
Source: Evid Based Complement Alternat Med. 2020 Sep 21;2020:8502530. doi: 10.1155/2020/8502530 (PMC7525299; doi:10.1155/2020/8502530)
Supplement: Supplementary Materials — The table contains detailed information about the review. [file 8502530.f1.docx]

| **Number** | **Author** | **Year** | **Nationality Distribution** | | | | | | | | | | **Classification of Participants** | | | **Group** | **Sample Size** | **Diseases** | | | | | | | | | | | | | | | | | | | **Gender** | | **Handedness** | **Acupuncture Experience** | **Emotional State** | | | | | | | | **Neuroimaging Technology** | | | | | **Manipulation Procedure** | **Qualification of Acupuncturists** | **Deqi** | **Acupuncture Modalities** | | | | | **Outcome Assessment**Evaluation of acupuncture sensation Ethical Review Acupoint selection |
| --- | --- | --- | --- | --- | --- | --- | --- | --- | --- | --- | --- | --- | --- | --- | --- | --- | --- | --- | --- | --- | --- | --- | --- | --- | --- | --- | --- | --- | --- | --- | --- | --- | --- | --- | --- | --- | --- | --- | --- | --- | --- | --- | --- | --- | --- | --- | --- | --- | --- | --- | --- | --- | --- | --- | --- | --- | --- | --- | --- | --- | --- | --- |
|  |  |  | China | Korea | Japan | UK | USA | Germany | Taiwan | Denmark | Italy | Multinational cooperation | healthy subjects | patients | HC+P |  |  | HC | Rheumatoid arthritis | Postoperative pain | Migraine | Cervical spondylosis | Primary dysmenorrhea | Peripheral facial paralysis | Pancreatitis | Low back pain | Endometriosis | Thalamic pain | Headache | Irritable bowel syndrome | Shoulder Pain | Knee osteoarthritis | Carpal tunnel syndrome | Sciatica | Fibromyalgia | Other | M | F |  |  | SDS | SAS | MMSE | STAI | BDI | PSQI | HAMD | claustrophobia | fMRI | PET | EEG | MEG | TCD |  |  |  | Manual acupuncture | TENS | Other | Auricular Acupuncture | Electroacupuncture | VASPROMIS-29 pain interference BPI NRS MPQ Pain threshold detector Duration of pain 0-10 Deqi Score VAS MASS NRS SASS 11-point Numerical Pain Rating NSQ specific acupoint standardized/semi-standard |
| 1 | Zhen Jin | 2001 | √ |  |  |  |  |  |  |  |  |  | √ |  |  | 1 | 11 |  |  |  |  |  |  |  |  |  |  |  |  |  |  |  |  |  |  |  | 6 | 5 |  | √ |  |  |  |  |  |  |  |  | √ |  |  |  |  | √ |  |  |  | √ |  |  |  | ST 36, SP 6 |
| 2 | Jinwen Hou | 2002 |  |  |  |  |  |  |  |  |  | China and USA | √ |  |  | 1 | 8 |  |  |  |  |  |  |  |  |  |  |  |  |  |  |  |  |  |  |  | 4 | 4 | √ |  |  |  |  |  |  |  |  |  | √ |  |  |  |  | √ | √ | √ | √ |  |  |  |  | LI 4 |
| 3 | Bo Liu | 2004 | √ |  |  |  |  |  |  |  |  |  |  | √ |  | 2 | 115（58/57） |  |  |  | √ |  |  |  |  |  |  |  |  |  |  |  |  |  |  |  | 34 | 81 |  |  |  |  |  |  |  |  |  |  |  |  |  |  | √ | √ |  | √ | √ |  |  |  |  | √√ √ |
| 4 | Weihong Zhong | 2004 | √ |  |  |  |  |  |  |  |  |  |  | √ |  | 2 | 40 |  |  |  |  | √ |  |  |  |  |  |  |  |  |  |  |  |  |  |  | 20 | 20 |  |  |  |  |  |  |  |  |  |  |  |  |  |  | √ | √ |  | √ | √ |  |  |  |  | √ √ √ √ |
| 5 | Junhai Zhang | 2005 | √ |  |  |  |  |  |  |  |  |  | √ |  |  | 1 | 18 |  |  |  |  |  |  |  |  |  |  |  |  |  |  |  |  |  |  |  | 10 | 8 | √ | naive |  |  |  |  |  |  |  |  | √ |  |  |  |  | √ |  | √ |  |  |  |  | √ | GB 34, GB 39 |
| 6 | Junhai Zhang | 2005 | √ |  |  |  |  |  |  |  |  |  | √ |  |  | 1 | 20 |  |  |  |  |  |  |  |  |  |  |  |  |  |  |  |  |  |  |  | 12 | 8 | √ |  |  |  |  |  |  |  |  |  | √ |  |  |  |  | √ |  |  |  |  |  |  | √ | √ GB 34, GB 39 |
| 7 | Ping Gong | 2006 | √ |  |  |  |  |  |  |  |  |  |  | √ |  | 1 | 6 |  |  |  |  |  | √ |  |  |  |  |  |  |  |  |  |  |  |  |  | 0 | 6 | √ |  |  |  |  |  |  |  |  |  |  | 18F-FDG |  |  |  | √ |  | √ | √ |  |  |  |  | √SP 6 |
| 8 | Xu Chi | 2007 | √ |  |  |  |  |  |  |  |  |  | √ |  |  | 1 | 6 |  |  |  |  |  |  |  |  |  |  |  |  |  |  |  |  |  |  |  | 3 | 3 | √ |  |  |  |  |  |  |  |  |  | √ |  |  |  |  | √ |  | √ | √ |  |  |  |  | SI 3, SJ 5, SJ 3 |
| 9 | Junzhou Han | 2007 | √ |  |  |  |  |  |  |  |  |  |  | √ |  | 3 | 36 |  |  |  |  |  |  | √ |  |  |  |  |  |  |  |  |  |  |  |  |  |  |  |  |  |  |  |  |  |  |  |  | √ |  |  |  |  | √ |  | √ |  |  |  |  | √ | √ |
| 10 | Aibing Li | 2008 | √ |  |  |  |  |  |  |  |  |  |  | √ |  | 2 | 160 |  |  |  | √ |  |  |  |  |  |  |  |  |  |  |  |  |  |  |  | 22 | 138 | √ |  |  |  |  |  |  |  |  |  |  |  |  |  | √ | √ |  |  | √ |  |  |  |  | √√ √ |
| 11 | Jijun Tong | 2008 | √ |  |  |  |  |  |  |  |  |  | √ |  |  | 3 | 12 |  |  |  |  |  |  |  |  |  |  |  |  |  |  |  |  |  |  |  |  |  | √ |  |  |  |  |  |  |  |  |  | √ |  | √ |  |  | √ |  |  |  |  | Manual+TENS |  |  | √ √ LI 4 |
|  |  |  |  |  |  |  |  |  |  |  |  |  |  |  |  | 1 | 8 |  |  |  |  |  |  |  |  |  |  |  |  |  |  |  |  |  |  |  | 4 | 4 | √ |  |  |  |  |  |  |  |  |  |  |  |  |  |  |  |  |  |  |  |  |  |  | √ |
| 12 | Junhai Zhang | 2008 | √ |  |  |  |  |  |  |  |  |  |  | √ |  | 1 | 12 |  |  |  |  |  |  |  |  | √ |  |  |  |  |  |  |  |  |  |  | 7 | 5 |  |  |  |  |  |  |  |  |  |  | √ |  |  |  |  | √ | √ | √ |  |  |  |  | √ | GB 34, GB 39 |
| 13 | Xuezhi Li | 2009 | √ |  |  |  |  |  |  |  |  |  |  | √ |  | 3 | 30 |  |  |  | √ |  |  |  |  |  |  |  |  |  |  |  |  |  |  |  | 12 | 18 | √ |  |  |  |  |  |  |  |  |  |  | 18F-FDG |  |  |  | √ | √ | √ |  |  |  |  | √ | √√ √ |
| 14 | Man Liao | 2010 | √ |  |  |  |  |  |  |  |  |  |  | √ |  | 2 | 52 |  |  |  | √ |  |  |  |  |  |  |  |  |  |  |  |  |  |  |  | 13 | 39 |  |  |  |  |  |  |  |  |  |  |  |  |  |  | √ | √ |  | √ |  |  |  |  | √ | √√ |
| 15 | Ming Lu | 2011 | √ |  |  |  |  |  |  |  |  |  |  | √ |  | 2 | 80 |  |  |  |  |  |  |  |  |  |  | √ |  |  |  |  |  |  |  |  | 51 | 29 |  |  |  |  |  |  |  |  |  |  |  |  |  |  | √ | √ |  | √ |  |  |  |  | √ | √ √ |
| 16 | Jie Yang | 2011 | √ |  |  |  |  |  |  |  |  |  |  | √ |  | 4 | 40 |  |  |  | √ |  |  |  |  |  |  |  |  |  |  |  |  |  |  |  | 16 | 24 | √ |  |  |  |  |  |  |  |  |  |  | 18F-FDG |  |  |  | √ | √ | √ |  |  |  |  | √ | √Yao ashi |
| 17 | Yongsong Ye | 2011 | √ |  |  |  |  |  |  |  |  |  |  |  | √ | 2 | 20 | 10 |  |  |  |  |  |  |  | √ |  |  |  |  |  |  |  |  |  |  | 4 | 6 |  |  |  |  |  |  |  |  |  |  | √ |  |  |  |  | √ | √ | √ | √ |  |  |  |  | √√ |
| 18 | Lei Zhang | 2011 | √ |  |  |  |  |  |  |  |  |  | √ |  |  | 1 | 14 |  |  |  |  |  |  |  |  |  |  |  |  |  |  |  |  |  |  |  | 7 | 7 | √ | √ |  |  |  |  |  |  |  |  | √ |  |  |  |  | √ |  |  |  | √ |  |  |  | √ ST 36, LR 3, GB 40, BL 63 |
| 19 | Yanhua Hu | 2013 | √ |  |  |  |  |  |  |  |  |  |  | √ |  | 2 | 80 |  |  |  |  | √ |  |  |  |  |  |  |  |  |  |  |  |  |  |  | 44 | 36 |  |  |  |  |  |  |  |  |  |  |  |  |  |  | √ | √ |  | √ |  |  | Manual+Tuina |  |  | √ |
| 20 | Liwei Xiao | 2013 | √ |  |  |  |  |  |  |  |  |  |  | √ |  | 2 | 76 |  |  |  |  |  |  |  |  |  |  |  | √ |  |  |  |  |  |  |  | 17 | 43 |  |  |  |  |  |  |  |  |  |  |  |  |  |  | √ | √ |  | √ | √ |  |  |  |  | √√ |
| 21 | Xiaolei Zhu | 2013 | √ |  |  |  |  |  |  |  |  |  |  | √ |  | 2 | 60 |  |  |  |  |  |  |  |  |  |  |  | √ |  |  |  |  |  |  |  | 16 | 44 |  |  |  |  |  |  |  |  |  |  |  |  |  |  | √ | √ |  | √ | √ |  |  |  |  | √√ |
| 22 | Ling Zhao | 2014 | √ |  |  |  |  |  |  |  |  |  | √ |  |  | 2 | 40 |  |  |  |  |  |  |  |  |  |  |  |  |  |  |  |  |  |  |  | 20 | 20 | √ | naive | √ | √ |  |  |  |  |  |  | √ |  |  |  |  | √ | √ | √ | √ |  |  |  |  | √ GB 40, PC 6, SJ 5, LR 3 |
| 23 | Hao Cheng | 2014 | √ |  |  |  |  |  |  |  |  |  | √ |  |  | 2 | 28 |  |  |  |  |  |  |  |  |  |  |  |  |  |  |  |  |  |  |  | 14 | 14 | √ | naive |  |  |  |  |  |  |  |  |  |  |  | √ |  | √ | √ | √ | √ |  |  |  |  | √ √ ST 36 |
| 24 | Yong Zhang | 2014 | √ |  |  |  |  |  |  |  |  |  |  | √ |  | 1 | 10 |  |  |  | √ |  |  |  |  |  |  |  |  |  |  |  |  |  |  |  | 2 | 8 | √ |  |  | √ | √ |  |  | √ | √ |  | √ |  |  |  |  | √ |  | √ | √ |  |  |  |  | √√ √ |
| 25 | Yi Ren | 2014 | √ |  |  |  |  |  |  |  |  |  |  | √ |  | 1 | 10 |  |  |  | √ |  |  |  |  |  |  |  |  |  |  |  |  |  |  |  | 2 | 8 | √ |  |  | √ | √ |  |  | √ | √ |  | √ |  |  |  |  | √ | √ | √ | √ |  |  |  |  | √√ √ GB 41 |
| 26 | Zhiming Zhang | 2014 | √ |  |  |  |  |  |  |  |  |  |  | √ |  | 3 | 113（37/38/38） |  |  |  | √ |  |  |  |  |  |  |  |  |  |  |  |  |  |  |  | 56 | 57 |  |  |  |  |  |  |  |  |  |  |  |  |  |  | √ | √ |  |  | √ |  |  |  |  | √√ |
| 27 | Xiangzhu Chen | 2015 | √ |  |  |  |  |  |  |  |  |  |  | √ |  | 1 | 6 |  |  |  |  |  | √ |  |  |  |  |  |  |  |  |  |  |  |  |  | 0 | 6 | √ |  | √ | √ |  |  |  |  |  |  |  | 18F-FDG |  |  |  | √ | √ | √ |  |  |  |  | √ | √SP 6 |
| 28 | Tao Guo | 2015 | √ |  |  |  |  |  |  |  |  |  |  | √ |  | 2 | 30（16/14） |  |  |  |  |  |  |  |  | √ |  |  |  |  |  |  |  |  |  |  | 14 | 16 | √ | √ |  |  |  |  |  |  |  |  | √ |  |  |  |  | √ |  | √ | √ |  |  |  |  | √√ √ |
| 29 | Shuhui Kan | 2015 | √ |  |  |  |  |  |  |  |  |  | √ |  |  | 3 | 9 |  |  |  |  |  |  |  |  |  |  |  |  |  |  |  |  |  |  |  | 4 | 5 |  |  |  |  |  |  |  |  |  |  |  |  |  | √ |  |  |  |  | √ |  |  |  |  | LI 4, ST 36 |
| 30 | Yanlin Xu | 2015 | √ |  |  |  |  |  |  |  |  |  |  | √ |  | 2 | 60 |  |  |  | √ |  |  |  |  |  |  |  |  |  |  |  |  |  |  |  | 20 | 40 |  |  |  |  |  |  |  |  |  |  |  |  |  |  | √ | √ |  | √ |  |  |  |  | √ | √√ |
| 31 | Chengguo Su | 2016 | √ |  |  |  |  |  |  |  |  |  |  | √ |  | 1 | 16 |  |  |  |  |  | √ |  |  |  |  |  |  |  |  |  |  |  |  |  | 0 | 16 | √ | √ |  |  |  |  |  |  |  |  | √ |  |  |  |  | √ | √ | √ | √ |  |  |  |  | √SP 6, SP 8 |
| 32 | Yongxia Wang | 2016 | √ |  |  |  |  |  |  |  |  |  |  | √ |  | 2 | 12 |  |  |  |  |  | √ |  |  |  |  |  |  |  |  |  |  |  |  |  | 0 | 12 | √ |  | √ | √ |  |  |  |  |  |  | √ |  |  |  |  | √ | √ | √ |  |  |  |  | √ | √SP 6 |
| 33 | Yurong Wen | 2016 | √ |  |  |  |  |  |  |  |  |  |  | √ |  | 2 | 40 |  |  |  |  |  |  |  |  | √ |  |  |  |  |  |  |  |  |  |  | 12 | 8 | √ |  | √ | √ |  |  |  |  |  |  | √ |  |  |  |  | √ | √ | √ |  |  |  |  | √ | √√ |
| 34 | Wenyuan Xie | 2016 | √ |  |  |  |  |  |  |  |  |  |  | √ |  | 2 | 38 |  |  |  | √ |  |  |  |  |  |  |  |  |  |  |  |  |  |  |  | 8 | 30 |  | √ | √ | √ |  |  |  |  |  |  | √ |  |  |  |  | √ |  | √ | √ |  |  |  |  | √Tou ashi |
| 35 | Yijun Liu | 2017 | √ |  |  |  |  |  |  |  |  |  |  | √ |  | 1 | 10 |  |  |  |  |  |  |  |  | √ |  |  |  |  |  |  |  |  |  |  | 5 | 5 | √ |  |  |  |  |  |  |  |  |  | √ |  |  |  |  | √ |  | √ | √ |  |  |  |  | √√ Gen ashi |
| 36 | Xing Wang | 2017 | √ |  |  |  |  |  |  |  |  |  |  |  | √ | 2 | 55 | 24 |  |  | √ |  |  |  |  |  |  |  |  |  |  |  |  |  |  |  | 0 | 55 | √ |  |  |  |  |  |  |  |  |  | √ |  |  |  |  | √ |  |  | √ |  |  |  |  | √√ |
| 37 | Liangda Yan | 2017 | √ |  |  |  |  |  |  |  |  |  |  | √ |  | 3 | 92（31/31/30） |  |  |  |  | √ |  |  |  |  |  |  |  |  |  |  |  |  |  |  | 44 | 48 |  |  |  |  |  |  |  |  |  |  |  |  |  |  | √ | √ |  | √ | √ |  |  |  |  | √ |
| 38 | Yuanyuan Chang | 2018 | √ |  |  |  |  |  |  |  |  |  |  | √ |  | 1 | 20 |  |  |  |  |  |  |  |  |  |  |  |  |  |  |  |  |  |  | Unspecified |  |  |  | naive |  |  |  |  |  |  |  |  |  |  | √ |  |  | √ |  |  |  | √ |  |  |  | PC 6, DU 20 |
| 39 | Yan Li | 2018 | √ |  |  |  |  |  |  |  |  |  |  | √ |  | 3 | 96 |  |  |  | √ |  |  |  |  |  |  |  |  |  |  |  |  |  |  |  | 39 | 57 |  |  |  |  |  |  |  |  |  |  |  |  |  |  | √ | √ |  | √ |  |  | Manual+Tuina |  |  | √ √ |
| 40 | Ziyi Yang | 2018 | √ |  |  |  |  |  |  |  |  |  |  | √ |  | 2 | 38（17/21） |  |  |  | √ |  |  |  |  |  |  |  |  |  |  |  |  |  |  |  | 0 | 38 | √ |  |  |  |  |  |  |  |  |  | √ |  |  |  |  | √ | √ | √ | √ |  |  |  |  | √√ √ |
| 41 | Shusen Zhang | 2018 | √ |  |  |  |  |  |  |  |  |  |  | √ |  | 2 | 27（13/14） |  |  |  | √ |  |  |  |  |  |  |  |  |  |  |  |  |  |  |  | 8 | 19 | √ |  |  |  |  |  |  |  |  |  | √ |  |  |  |  | √ |  | √ | √ |  |  |  |  | √√ √ |
| 42 | Siyang Zhao | 2018 | √ |  |  |  |  |  |  |  |  |  |  | √ |  | 2 | 60 |  |  |  | √ |  |  |  |  |  |  |  |  |  |  |  |  |  |  |  | 22 | 38 |  |  |  |  |  |  |  |  |  |  |  |  |  |  | √ | √ |  | √ |  |  |  |  | √ | √√ |
| 43 | M T Wu | 1999 |  |  |  |  |  |  | √ |  |  |  | √ |  |  | 2 | 18 |  |  |  |  |  |  |  |  |  |  |  |  |  |  |  |  |  |  |  | 11 | 7 | √ |  |  |  |  |  |  |  |  |  | √ |  |  |  |  | √ | √ |  | √ |  |  |  |  | √ √ ST 36, LI 4 |
| 44 | [G Biella](https://doi.org/10.1006/nimg.2001.0798) | 2001 |  |  |  |  |  |  |  |  | √ |  | √ |  |  | 2 | 13 |  |  |  |  |  |  |  |  |  |  |  |  |  |  |  |  |  |  |  | 13 | 0 |  |  |  |  |  |  |  |  |  |  |  | H215O |  |  |  | √ |  | √ | √ |  |  |  |  | √ ST 36, LU 5 |
| 45 | [JC Hsieh](https://doi.org/10.1016/s0304-3940(01)01952-8) | 2001 |  |  |  |  |  |  | √ |  |  |  | √ |  |  | 1 | 16 |  |  |  |  |  |  |  |  |  |  |  |  |  |  |  |  |  |  |  | 8 | 8 | √ |  |  |  |  |  |  |  |  |  |  | H215O |  |  |  | √ |  | √ | √ |  |  |  |  | √ LI 4 |
| 46 | [Ming-Ting Wu](https://doi.org/10.1006/nimg.2002.1145) | 2002 |  |  |  |  |  |  | √ |  |  |  | √ |  |  | 1 | 15 |  |  |  |  |  |  |  |  |  |  |  |  |  |  |  |  |  |  |  | 10 | 5 | √ | naive |  |  |  |  |  |  |  |  | √ |  |  |  |  | √ | √ | √ |  |  |  |  | √ | √ √ GB 34 |
| 47 | [Jérémie Pariente](https://doi.org/10.1016/j.neuroimage.2005.01.016) | 2005 |  |  |  | √ |  |  |  |  |  |  |  | √ |  | 1 | 14 |  |  |  |  |  |  |  |  |  |  |  |  |  |  |  |  |  |  | Phalangeal osteoarthritis | 3 | 11 | √ | √ |  |  |  |  |  |  |  |  |  | H215O |  |  |  | √ |  | √ | √ |  |  |  |  | √√ √ √ LI 4 |
| 48 | Jian Kong | 2006 |  |  |  |  | √ |  |  |  |  |  | √ |  |  | 1 | 24 |  |  |  |  |  |  |  |  |  |  |  |  |  |  |  |  |  |  |  | 13 | 11 | √ | naive |  |  |  |  |  |  |  |  | √ |  |  |  |  |  |  |  | √ |  |  |  |  | LI 4, SI 3 |
| 49 | [Vitaly Napadow](https://doi.org/10.1002/hbm.20261) | 2007 |  |  |  |  | √ |  |  |  |  |  |  |  | √ | 1 | 19 | 9 |  |  |  |  |  |  |  |  |  |  |  |  |  |  | √ |  |  |  | 7 | 12 |  |  |  |  |  |  |  |  |  |  | √ |  |  |  |  |  |  |  |  |  |  |  | √ | √ √ |
| 50 | [Wei Qin](https://doi.org/10.1186/1744-8069-4-55) | 2008 | √ |  |  |  |  |  |  |  |  |  | √ |  |  | 1 | 18 |  |  |  |  |  |  |  |  |  |  |  |  |  |  |  |  |  |  |  | 9 | 9 | √ | naive |  |  |  |  |  |  |  |  | √ |  |  |  |  | √ |  |  | √ |  |  |  |  | √ ST 36 |
| 51 | [Richard E Harris](https://doi.org/10.1016/j.neuroimage.2009.05.083) | 2009 |  |  |  | √ |  |  |  |  |  |  |  | √ |  | 2 | 20 |  |  |  |  |  |  |  |  |  |  |  |  |  |  |  |  |  | √ |  | 0 | 20 | √ | naive |  |  |  |  |  |  |  |  |  | 11C-carfentanil |  |  |  |  |  |  |  |  | Manual+Auricular |  |  | √ √ √ |
| 52 | [Jian Kong](https://doi.org/10.1016/j.neuroimage.2008.12.025) | 2009 |  |  |  |  | √ |  |  |  |  |  | √ |  |  | 4 | 48 |  |  |  |  |  |  |  |  |  |  |  |  |  |  |  |  |  |  |  | 24 | 24 | √ | naive |  |  |  |  |  |  |  |  | √ |  |  |  |  | √ | √ |  |  |  |  |  | √ | √ LI 4, LI 3 |
| 53 | [Shivshil Shukla](https://doi.org/10.1186/1744-8069-7-45) | 2011 |  |  |  |  | √ |  |  |  |  |  | √ |  |  | 1 | 10 |  |  |  |  |  |  |  |  |  |  |  |  |  |  |  |  |  |  |  | 4 | 6 | √ |  |  |  |  |  |  |  |  | √ | √ |  |  |  |  | √ | √ | √ |  |  |  |  | √ | √ √ LR1, SP 1 |
| 54 | Ji Li | 2012 | √ |  |  |  |  |  |  |  |  |  |  | √ |  | 2 | 20 |  |  |  |  |  |  |  |  |  |  |  |  |  |  |  |  | √ |  |  | 10 | 10 |  |  |  |  |  |  |  |  |  |  | √ |  |  |  |  | √ |  |  |  |  |  |  | √ | √√ |
| 55 | [Javeria Ali Hashmi](https://doi.org/10.1523/jneurosci.3155-13.2014) | 2014 |  |  |  |  | √ |  |  |  |  |  |  | √ |  | 2 | 42 |  |  |  |  |  |  |  |  |  |  |  |  |  |  | √ |  |  |  |  | 17 | 25 |  |  |  |  |  | √ | √ |  |  |  | √ |  |  |  |  | √ | √ |  |  |  |  |  | √ | √ √ LI 4, LI 3 |
| 56 | Nina Theysohn | 2014 |  |  |  |  |  | √ |  |  |  |  | √ |  |  | 1 | 14 |  |  |  |  |  |  |  |  |  |  |  |  |  |  |  |  |  |  |  | 7 | 7 |  |  |  |  |  |  |  |  |  |  | √ |  |  |  |  | √ |  | √ | √ |  |  |  |  | √ √ LI 4, ST 36, LR 3 |
| 57 | Ling Zhao | 2014 | √ |  |  |  |  |  |  |  |  |  |  | √ |  | 2 | 40 |  |  |  | √ |  |  |  |  |  |  |  |  |  |  |  |  |  |  |  | 14 | 26 | √ |  |  |  |  |  |  |  |  |  | √ |  |  |  |  | √ | √ | √ | √ |  |  |  |  | √√ √ |
| 58 | Natalia Egorova | 2015 |  |  |  |  | √ |  |  |  |  |  |  | √ |  | 3 | 30 |  |  |  |  |  |  |  |  |  |  |  |  |  |  | √ |  |  |  |  | 17 | 13 |  |  |  |  |  |  |  |  |  |  | √ |  |  |  |  |  |  | √ | √ |  |  |  |  | √ √ |
| 59 | Kuangshi Li | 2015 | √ |  |  |  |  |  |  |  |  |  |  |  | √ | 2 | 24 | 12 |  |  | √ |  |  |  |  |  |  |  |  |  |  |  |  |  |  |  | 4 | 20 | √ | naive |  |  |  |  |  |  |  |  | √ |  |  |  |  | √ | √ | √ | √ |  |  |  |  | √ √ |
| 60 | Taras I Usichenko | 2015 |  |  |  |  |  | √ |  |  |  |  | √ |  |  | 1 | 21 |  |  |  |  |  |  |  |  |  |  |  |  |  |  |  |  |  |  |  | 21 | 0 | √ |  |  |  |  |  |  |  |  |  | √ |  |  |  |  | √ |  |  | √ |  |  |  |  | √ √ ST 44 |
| 61 | Zhengjie Li | 2016 | √ |  |  |  |  |  |  |  |  |  |  |  | √ | 5 | 104 | 62 |  |  | √ |  |  |  |  |  |  |  |  |  |  |  |  |  |  |  | 14 | 48 | √ | √ |  |  |  |  |  |  |  | √ | √ |  |  |  |  | √ | √ | √ | √ |  |  |  |  | √√ √ |
| 62 | Jixin Liu | 2017 | √ |  |  |  |  |  |  |  |  |  |  |  | √ | 3 | 121（50/50/121） | 50 |  |  | √ |  |  |  |  |  |  |  |  |  |  |  |  |  |  |  |  |  | √ |  |  |  |  |  |  |  |  | √ | √ |  |  |  |  |  |  |  | √ |  |  |  |  | √√ √ |
| 63 | Yumi Maed | 2017 |  |  |  |  |  |  |  |  |  | USA and Korea |  |  | √ |  | 114 | 34 |  |  |  |  |  |  |  |  |  |  |  |  |  |  | √ |  |  |  | 21 | 92 |  | √ |  |  |  |  |  |  |  |  | √ |  |  |  |  | √ | √ | √ | √ |  |  |  |  | √ √ |
| 64 | Meena M Makary | 2018 |  |  |  |  |  |  |  |  |  | USA and Korea |  | √ |  | 2 | 56（33/23） |  |  |  |  |  |  |  |  | √ |  |  |  |  |  |  |  |  |  |  | 31 | 25 |  |  |  |  |  |  |  |  |  |  | √ |  |  |  |  | √ | √ | √ | √ |  |  |  |  | √√ √ |
| 65 | Yan Zou | 2019 | √ |  |  |  |  |  |  |  |  |  |  |  | √ | 1 | 32 | 18 |  |  | √ |  |  |  |  |  |  |  |  |  |  |  |  |  |  |  | 14 | 18 |  | √ |  |  |  |  |  |  |  |  | √ |  |  |  |  | √ |  | √ | √ |  |  |  |  | √√ √ |
| 66 | V Napadow | 2007 |  |  |  |  | √ |  |  |  |  |  |  |  | √ | 1 | 19（9/10） | 9 |  |  |  |  |  |  |  |  |  |  |  |  |  |  | √ |  |  |  | 7 | 12 |  |  |  |  |  |  |  |  |  |  | √ |  |  |  |  |  | √ | √ |  |  |  |  | √ | √√ LI 4 |
| 67 | Xiaoyan Chen | 2015 |  |  |  |  | √ |  |  |  |  |  |  | √ |  | 3 | 30（10/10/10） |  |  |  |  |  |  |  |  |  |  |  |  |  |  | √ |  |  |  |  | 17 | 13 |  | naive |  |  |  |  |  |  |  |  | √ |  |  |  |  | √ |  | √ | √ |  |  |  |  | √ √ √ √ |
| 68 | Jian Kong | 2018 |  |  |  | √ |  |  |  |  |  |  |  | √ |  | 3 | 46（17/12/17） |  |  |  |  |  |  |  |  |  |  |  |  |  |  | √ |  |  |  |  | 27 | 19 |  |  |  |  |  |  |  |  |  |  | √ |  |  |  |  | √ |  | √ | √ |  |  |  |  | √√ √ √ |
| 69 | Hyungjun Kim | 2020 |  |  |  |  |  |  |  |  |  | USA and Korea |  |  | √ | 4 | 152（18/18/19/23） | 74 |  |  |  |  |  |  |  | √ |  |  |  |  |  |  |  |  |  |  | 60 | 92 |  |  |  |  |  |  |  |  |  |  | √ |  |  |  |  | √ | √ |  | √ |  |  |  |  | √ √ √ √ |
| 70 | Siyi Yu | 2020 |  |  |  |  | √ |  |  |  |  |  |  | √ |  | 4 | 50（14/13/14/13） |  |  |  |  |  |  |  |  | √ |  |  |  |  |  |  |  |  |  |  | 19 | 31 |  |  |  |  |  |  |  |  |  | √ | √ |  |  |  |  | √ | √ | √ | √ |  |  |  |  | √ √ √ |
| 71 | Ching-Hsiung Liu | 2020 |  |  |  |  |  |  | √ |  |  |  |  |  | √ | 2 | 27（12/15） | 15 |  |  |  |  |  |  |  |  |  |  |  |  |  |  |  | √ |  |  | 6 | 6 |  |  |  |  |  |  |  |  |  |  | √ |  |  |  |  | √ | √ | √ | √ |  |  |  |  | √√ √ |
| 72 | Chao-Qun Yan | 2020 | √ |  |  |  |  |  |  |  |  |  |  | √ |  | 2 | 24（12/12） |  |  |  |  |  |  |  |  |  |  |  |  |  | √ |  |  |  |  |  |  |  |  |  |  |  |  |  |  |  |  |  | √ |  |  |  |  |  |  | √ | √ |  |  |  |  | √√ ST 38 |
| 73 | Jeungchan Lee | 2019 |  |  |  |  |  |  |  |  |  | USA and Korea |  | √ |  | 2 | 43（25/18） |  |  |  |  |  |  |  |  | √ |  |  |  |  |  |  |  |  |  |  | 21 | 22 |  | √ |  |  |  |  |  |  |  |  | √ |  |  |  |  | √ | √ | √ | √ |  |  |  |  | √√ √ |
| 74 | Anfeng Xiang | 2019 | √ |  |  |  |  |  |  |  |  |  |  | √ |  | 1 | 12 |  |  |  |  |  |  |  |  | √ |  |  |  |  |  |  |  |  |  |  | 7 | 5 | √ |  |  |  |  |  |  |  |  | √ | √ |  |  |  |  | √ | √ |  | √ |  |  |  |  | √√ √ √ |
| 75 | Yiheng Tu | 2019 |  |  |  |  | √ |  |  |  |  |  |  | √ |  | 4 | 50（14/13/14/13） |  |  |  |  |  |  |  |  | √ |  |  |  |  |  |  |  |  |  |  | 19 | 31 |  |  |  |  |  |  |  |  |  |  | √ |  |  |  |  | √ | √ | √ | √ |  |  |  |  | √√ √ |
| 76 | Jixin Liu | 2019 | √ |  |  |  |  |  |  |  |  |  |  | √ |  | 2 | 94（38/56） |  |  |  | √ |  |  |  |  |  |  |  |  |  |  |  |  |  |  |  |  |  |  | √ |  |  |  |  |  |  |  | √ | √ |  |  |  |  | √ |  | √ | √ |  |  |  |  | √ √ √ |
| 77 | Danielle M Graff | 2018 |  |  |  |  | √ |  |  |  |  |  |  | √ |  | 1 | 19 |  |  |  | √ |  |  |  |  |  |  |  |  |  |  |  |  |  |  |  | 2 | 17 |  |  |  |  |  |  |  |  |  |  | √ |  |  |  |  | √ | √ |  |  |  |  | √ |  | √√ √ |
| 78 | Randy L Gollub | 2018 |  |  |  |  | √ |  |  |  |  |  |  | √ |  | 2 | 43（21/22） |  |  |  |  |  |  |  |  |  |  |  |  |  |  | √ |  |  |  |  | 17 | 26 |  | √ |  |  |  |  |  |  |  |  | √ |  |  |  |  | √ | √ |  |  |  |  |  | √ | √ LI 4, LI 3 |
| 79 | Florian Beissner | 2018 |  |  |  |  |  | √ |  |  |  |  |  | √ |  | 2 | 60（30/30） |  |  |  |  |  |  |  |  |  | √ |  |  |  |  |  |  |  |  |  |  |  |  |  |  |  |  |  |  |  |  |  | √ |  |  |  |  |  | √ |  | √ |  |  |  |  | √ √ RN 3 |
| 80 | Xuan Niu | 2017 | √ |  |  |  |  |  |  |  |  |  | √ |  |  | 2 | 32（16/16） | √ |  |  |  |  |  |  |  |  |  |  |  |  |  |  |  |  |  |  | 16 | 16 | √ | √ |  |  |  |  |  |  |  |  | √ |  |  |  |  | √ |  | √ |  |  |  |  | √ | √ √ √ LI 4 |
| 81 | Jixin Liu | 2017 | √ |  |  |  |  |  |  |  |  |  |  |  | √ | 2 | 130（21/59） | 50 |  |  | √ |  |  |  |  |  |  |  |  |  |  |  |  |  |  |  |  |  | √ | naive |  |  |  |  |  |  |  | √ | √ |  |  |  |  |  |  | √ | √ |  |  |  |  | √ √ √ |
| 82 | Zhengjie Li | 2017 |  |  |  |  |  |  |  |  |  | China and USA |  | √ |  | 5 | 62（11/11/11/13/16） |  |  |  | √ |  |  |  |  |  |  |  |  |  |  |  |  |  |  |  | 14 | 48 | √ | √ |  |  |  |  |  |  |  | √ | √ |  |  |  |  | √ | √ | √ | √ |  |  |  |  | √√ √ √ |
| 83 | Jacob Juel | 2017 |  |  |  |  |  |  |  | √ |  |  |  | √ |  | 2 | 15（15/15） |  |  |  |  |  |  |  | √ |  |  |  |  |  |  |  |  |  |  |  | 8 | 7 |  |  |  |  |  |  |  |  |  |  |  |  | √ |  |  | √ | √ | √ | √ |  |  |  |  | √√ √ |
| 84 | Yong Zhang | 2016 | √ |  |  |  |  |  |  |  |  |  |  |  | √ | 2 | 24（12/12） | 12 |  |  | √ |  |  |  |  |  |  |  |  |  |  |  |  |  |  |  | 4 | 20 | √ |  |  |  |  |  |  |  |  |  | √ |  |  |  |  | √ | √ | √ | √ |  |  |  |  | √√ √ |
| 85 | Juan Xiao | 2016 | √ |  |  |  |  |  |  |  |  |  | √ |  |  | 1 | 15 | √ |  |  |  |  |  |  |  |  |  |  |  |  |  |  |  |  |  |  | 8 | 7 | √ | naive |  |  |  |  |  |  |  |  | √ |  |  |  |  | √ | √ |  |  |  |  |  | √ | √ √ GB 39, HT 5 |
| 86 | Zhao, J. M. | 2015 |  |  |  |  |  |  |  |  |  | China and USA |  | √ |  | 2 | 60（30/30） |  |  |  |  |  |  |  |  |  |  |  |  | √ |  |  |  |  |  |  |  |  |  |  |  |  |  |  |  |  |  |  | √ |  |  |  |  | √ |  | √ |  |  |  |  | √ | √√ √ |
| 87 | Taras I Usichenko | 2015 |  |  |  |  |  | √ |  |  |  |  | √ |  |  | 1 | 21 | √ |  |  |  |  |  |  |  |  |  |  |  |  |  |  |  |  |  |  |  |  | √ |  |  |  |  |  |  |  |  |  | √ |  |  |  |  | √ |  |  | √ |  |  |  |  | √ ST 44 |
| 88 | Yu Shi | 2015 | √ |  |  |  |  |  |  |  |  |  | √ |  |  | 1 | 28 | √ |  |  |  |  |  |  |  |  |  |  |  |  |  |  |  |  |  |  | 17 | 11 |  | √ |  |  |  |  |  |  |  |  | √ |  |  |  |  | √ |  | √ | √ |  |  |  |  | √ √ BL 40 |
| 89 | Natalia Egorova | 2015 |  |  |  |  | √ |  |  |  |  |  |  | √ |  | 3 | 30（10/10/10） |  |  |  |  |  |  |  |  |  |  |  |  |  |  | √ |  |  |  |  | 17 | 13 |  |  |  |  |  |  |  |  |  |  | √ |  |  |  |  | √ |  | √ | √ |  |  |  |  | √ √ √ |
| 90 | Yanqing Chen | 2015 | √ |  |  |  |  |  |  |  |  |  |  | √ |  | 2 | 60（30/30） |  |  | √ |  |  |  |  |  |  |  |  |  |  |  |  |  |  |  |  |  |  |  | √ |  |  |  |  |  |  |  |  | √ |  |  |  |  | √ |  | √ |  | √ |  |  |  | √ √ LI 4, ST 44 |
| 91 | Yumi Maeda | 2013 |  |  |  |  | √ |  |  |  |  |  |  | √ |  | 3 | 59（22/18/19） |  |  |  |  |  |  |  |  |  |  |  |  |  |  |  | √ |  |  |  | 10 | 49 |  |  |  |  |  |  |  |  |  |  | √ |  |  |  |  | √ |  | √ |  |  |  |  | √ | √√ SP 6, PC 7, SI 11 |
| 92 | Na-Hee Kim | 2013 |  | √ |  |  |  |  |  |  |  |  | √ |  |  | 1 | 20 | √ |  |  |  |  |  |  |  |  |  |  |  |  |  |  |  |  |  |  | 0 | 20 | √ | √ |  |  |  |  |  |  |  |  | √ |  |  |  |  | √ | √ |  | √ |  |  |  |  | √ √ BL 60 |
| 93 | Yongsong Ye | 2012 | √ |  |  |  |  |  |  |  |  |  |  | √ |  | 2 | 20（10/10） |  |  |  |  |  |  |  |  | √ |  |  |  |  |  |  |  |  |  |  | 8 | 12 | √ |  |  |  |  |  |  |  |  |  | √ |  |  |  |  | √ | √ | √ | √ |  |  |  |  | √√ √ |
| 94 | [Jie Yang](https://pubmed.ncbi.nlm.nih.gov/?term=Yang+J&cauthor_id=22894176) | 2012 | √ |  |  |  |  |  |  |  |  |  |  | √ |  | 3 | 30（10/10/10） |  |  |  | √ |  |  |  |  |  |  |  |  |  |  |  |  |  |  |  | 12 | 18 | √ |  |  |  |  |  |  |  |  |  |  | 18F-FDG |  |  |  | √ | √ | √ |  |  |  |  | √ | √√ √ |
| 95 | Bert Wetzel | 2011 |  |  |  |  |  | √ |  |  |  |  |  | √ |  | 2 | 120（60/60） |  |  | √ |  |  |  |  |  |  |  |  |  |  |  |  |  |  |  |  | 50 | 70 |  | √ |  |  |  |  |  |  |  |  | √ |  |  |  |  | √ |  |  |  |  |  | √ |  | √ √ |
| 96 | Carolyn E Zyloney | 2010 |  |  |  |  | √ |  |  |  |  |  | √ |  |  | 4 | 48（12/12/12/12） | √ |  |  |  |  |  |  |  |  |  |  |  |  |  |  |  |  |  |  | 24 | 24 | √ | naive |  |  |  |  |  |  |  |  | √ |  |  |  |  | √ | √ | √ |  |  |  |  | √ | √ √ LI 4,LI 3 |
| 97 | Darin D Dougherty | 2008 |  |  |  |  | √ |  |  |  |  |  | √ |  |  | 1 | 22 | √ |  |  |  |  |  |  |  |  |  |  |  |  |  |  |  |  |  |  | 12 | 10 | √ | naive |  |  |  |  |  |  |  |  | √ | [11C]diprenorphine |  |  |  | √ |  | √ | √ |  |  |  |  | √ √ LI 4 |
| 98 | W Meissner | 2004 |  |  |  |  |  | √ |  |  |  |  | √ |  |  | 2 | 16（8/8） | √ |  |  |  |  |  |  |  |  |  |  |  |  |  |  |  |  |  |  |  |  |  |  |  |  |  |  |  |  |  |  |  |  | √ |  |  | √ | √ |  |  |  |  |  | √ | √ ST 36, SP 6, LR 3 |
| 99 | Yiheng Tu | 2019 |  |  |  |  | √ |  |  |  |  |  |  | √ |  | 4 | 50（12/12/13/13） |  |  |  |  |  |  |  |  | √ |  |  |  |  |  |  |  |  |  |  | 19 | 31 |  |  |  |  |  |  |  |  |  |  | √ |  |  |  |  | √ | √ | √ | √ |  |  |  |  | √√ √ |
| 100 | W W Peng | 2019 | √ |  |  |  |  |  |  |  |  |  | √ |  |  | 4 | 80（20/20/20/20） | √ |  |  |  |  |  |  |  |  |  |  |  |  |  |  |  |  |  |  | 40 | 40 | √ | naive |  |  |  |  |  |  |  |  |  |  | √ |  |  | √ |  |  |  | √ |  |  |  | √ √ √ |
| 101 | Hauck, M. | 2017 |  |  |  |  |  | √ |  |  |  |  | √ |  |  | 1 | 26 | √ |  |  |  |  |  |  |  |  |  |  |  |  |  |  |  |  |  |  | 7 | 19 | √ |  |  |  |  |  |  |  |  | √ |  |  | √ |  |  | √ | √ |  | √ |  |  |  |  | √ √ √ |
| 102 | Jacob Juel | 2016 |  |  |  |  |  |  |  |  |  | Denmark and Italy | √ |  |  | 1 | 15 | √ |  |  |  |  |  |  |  |  |  |  |  |  |  |  |  |  |  |  | 7 | 8 |  |  |  |  |  |  |  |  |  |  |  |  | √ |  |  | √ | √ |  | √ |  |  |  |  | √ √ |
| 103 | Jacob Juel | 2017 |  |  |  |  |  |  |  |  |  | Denmark and Italy | √ |  |  | 2 | 16 | √ |  |  |  |  |  |  |  |  |  |  |  |  |  |  |  |  |  |  | 8 | 9 |  |  |  |  |  |  |  |  |  |  |  |  | √ |  |  | √ | √ |  | √ |  |  |  |  | √ √ |
| 104 | Ji Li | 2014 | √ |  |  |  |  |  |  |  |  |  |  |  | √ | 1 | 30 | 10 |  |  |  |  |  |  |  | √ |  |  |  |  |  |  |  |  |  |  | 15 | 15 | √ | naive |  |  |  |  |  |  |  |  | √ |  |  |  |  | √ | √ | √ | √ |  |  |  |  | √√ √ |
| 105 | Chuanfu Li | 2014 | √ |  |  |  |  |  |  |  |  |  | √ |  |  | 1 | 40 | √ |  |  |  |  |  |  |  |  |  |  |  |  |  |  |  |  |  |  | 20 | 20 | √ | √ |  |  |  |  |  |  |  |  | √ |  |  |  |  | √ |  | √ | √ |  |  |  |  | √ √ |
| 106 | Masami Sato | 2009 |  |  | √ |  |  |  |  |  |  |  |  | √ |  | 1 | 9 |  | √ |  |  |  |  |  |  |  |  |  |  |  |  |  |  |  |  |  |  |  |  | √ |  |  |  |  |  |  |  |  |  | 18F-FDG |  |  |  | √ | √ |  | √ |  |  |  |  | √√ √ |
| 107 | WT Zhang | 2003 | √ |  |  |  |  |  |  |  |  |  | √ |  |  | 2 | 48（24/24） | √ |  |  |  |  |  |  |  |  |  |  |  |  |  |  |  |  |  |  | 23 | 25 | √ |  |  |  |  |  |  |  |  |  | √ |  |  |  |  | √ |  |  |  |  |  |  | √ | √ ST 36 |
|  |  |  |  |  |  |  |  |  |  |  |  |  |  |  |  |  |  |  |  |  |  |  |  |  |  |  |  |  |  |  |  |  |  |  |  |  |  |  |  |  |  |  |  |  |  |  |  |  |  |  |  |  |  |  |  |  |  |  |  |  |  |  |
